# Comparison of outcomes between anticoagulation and antiplatelet therapies for intracranial arterial dissections

**DOI:** 10.3389/fneur.2024.1469697

**Published:** 2024-12-04

**Authors:** Seong-Joon Lee, Min Kim, So Young Park, Ji Hyun Park, Bumhee Park, Woo Sang Jung, Jin Wook Choi, Yong Cheol Lim, Ji Man Hong, Jin Soo Lee

**Affiliations:** ^1^Department of Neurology, Ajou University School of Medicine, Suwon, Republic of Korea; ^2^Office of Biostatistics, Medical Research Collaborating Center, Ajou Research Institute for Innovative Medicine, Ajou University Medical Center, Suwon, Republic of Korea; ^3^Department of Biomedical Informatics, Ajou University School of Medicine, Suwon, Republic of Korea; ^4^Department of Radiology, Ajou University School of Medicine, Suwon, Republic of Korea; ^5^Department of Neurosurgery, Ajou University School of Medicine, Suwon, Republic of Korea

**Keywords:** intracranial dissection, ischemic stroke, subarachnoid hemorrhage, anticoagulation, antiplatelet

## Abstract

**Background:**

This study aimed to evaluate real-world data on the differences in outcomes between antiplatelet (AP) and anticoagulation (AC) therapies for intracranial arterial dissection (IAD).

**Methods:**

This study included patients with symptomatic unruptured IAD between 2010 and 2021 that were treated with anti-thrombotics. Patients were dichotomized to AC and AP based on a treatment policy analysis. Primary endpoints were a composite of ischemic early neurological deterioration, recurrent ischemic or hemorrhagic stroke, or 3-month mortality. Arterial changes were evaluated both in the early (during admission) and late (after discharge) periods. A treatment effectiveness analysis was also performed with AC, AP and a third group of antithrombotic cross-overs. Propensity score matching (PSM) was used to adjust significant baseline differences.

**Results:**

In unruptured IAD patients (*N* = 311), the AC group (*N* = 211) presented with a higher rate of ischemic stroke or TIA (74.4% vs. 51.0%, *p* < 0.001) and steno-occlusive morphology (vs. dilatation, 63.0% vs. 39.0%, *p* < 0.001) compared to AP group (*N* = 100). After PSM, there was no difference in rates of primary endpoint (9.4% vs. 6.5%, *p* = 0.470). The results of the treatment effectiveness analysis resembled that of the treatment policy analysis. However, there was a high rate of cross-overs from AC to AP (57/211 [27.0%]). In this group, there was a higher rate of early arterial changes (26.8% vs. 13.1%, *p* = 0.019) compared to the AC group.

**Conclusion:**

In patients with unruptured IAD, this study did not show differences in primary endpoints according to antithrombotic regimen, while there was a high rate of cross-overs from AC to AP.

## Background and aims

In intracranial arterial dissections (IAD), the evidence with the use of antithrombotic agents is limited (1). For its counterpart, cervical arterial dissections (CAD), the Cervical Artery Dissection in Stroke Study (CADISS) failed to show differences in ipsilateral stroke or death, and angiographic recanalization rates between anticoagulation (AC) and antiplatelets (AP) (2). In addition, a more recent randomized trial (TREAT-CAD) did not show non-inferiority of aspirin to vitamin K antagonists (3). Based on the results from CADISS and TREAT-CAD, the use of aspirin as the standard therapy over anticoagulation in CAD patients is weak (1), and both anticoagulants and antiplatelets have been prescribed (4).

While the evidence for use of antithrombotic agents in IAD is limited, experts feel that AP have a better risk/benefit ratio over AC due to risk for subarachnoid hemorrhage (SAH) (4). In cases where endovascular reconstructive or deconstructive therapy (5) is planned to prevent rupture of dissecting aneurysms, AP would also be preferred. However, clinicians may still prefer to use AC to prevent embolization from fresh thrombus (2) and to promote recanalization or arterial healing. Evidence regarding such questions is lacking.

This study aimed to compare the differences in outcomes between antithrombotic modalities (AC vs. AP therapy) in terms of combined hemorrhagic and ischemic clinical outcomes and arterial outcomes.

## Methods

**Figure 1:**
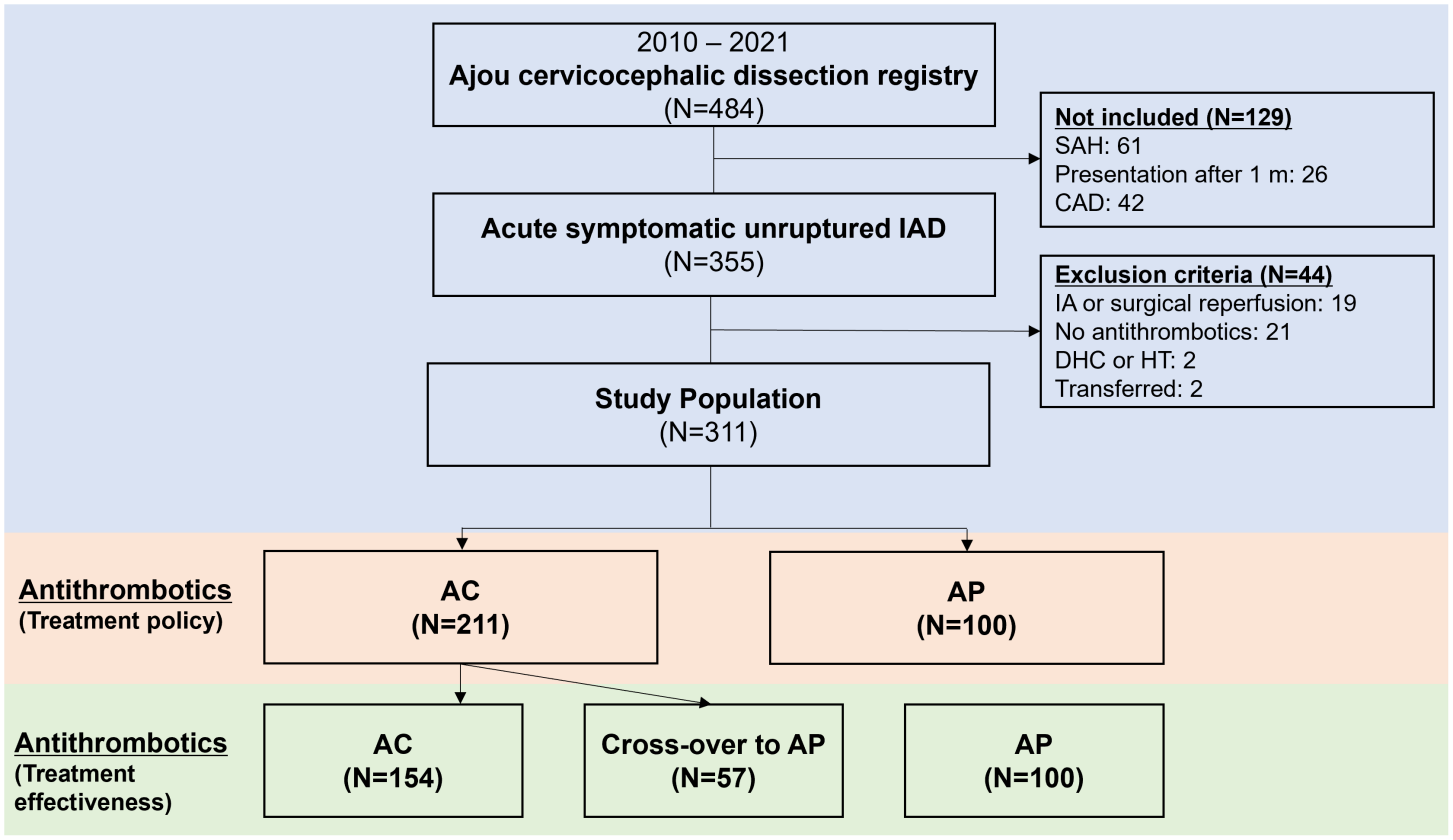
Flowchart of patients included in the current study. IAD, intracranial arterial dissections; SAH, subarachnoid hemorrhage; CAD, cervical arterial dissections; IA, intra-arterial; DHC, decompressive hemicraniectomy; HT, hemorrhagic transformation; AC, anticoagulation; AP, antiplatelets.

Patients that presented with symptoms due to acute intracranial arterial dissections and used antithrombotics were enrolled from an institutional registry of cervicocephalic dissections (6). The diagnosis of intracranial dissections was based on the presence of below imaging findings (7–9) involving the intracranial arteries; luminal pearl and string sign (stenosis and dilatation); luminal stenosis with intimal flap/double lumen, or intramural hematoma (at-suppression T1-weighted MR or MR angiogram source images); luminal fusiform aneurysmal dilatation of the arterial trunk not located at an arterial branching point; luminal occlusion with visible intimal flap/double lumen, or associated with a pearl-and-string sign. From this registry, patients who presented between 2010 and 2021 with onset of symptoms to presentation within 31 days, diagnosed with IAD without SAH, and prescribed antithrombotics for its treatment were selected. Patients were excluded if (1) they underwent emergent endovascular or surgical reperfusion, (2) did not use anti-thrombotic medication during hospitalization and at discharge due to any reason; (3) underwent hemicraniectomy or suffered significant hemorrhagic transformation limiting use of antithrombotics, or (4) were transferred to another hospital (Figure 1). Ethics approval was obtained from the Ajou University Hospital International Review Board (AJOUIRB-MDB-2021-674), and the study was performed in accordance with the ethical standards of the 1964 Declaration of Helsinki and its later amendments. The board waived the need for obtaining patient consent due to retrospective study nature.

### Definition of anticoagulation and antiplatelet therapy

Patients were dichotomized into the AC and AP groups. AC was performed by initiation of intravenous heparin (10) followed by warfarin, or warfarin alone. Patients that used a combination of AC and APs were included into the AC group. Use of IV argatroban (11) was not considered anticoagulation. Novel oral anticoagulants were not used. Patients that used only antiplatelets (usually immediate antiplatelet therapy) (12) were included in the AP group. This included aspirin, clopidogrel, cilostazol, triflusal, ticlopidine, or its combinations. The duration of anticoagulation was guided by repeat arterial imaging. It was usually used for 3 months to not more than 6 months, then switched to antiplatelets. In patients presenting with cerebral ischemia, the duration of antiplatelet therapy was lifelong. If the patient did not present with ischemia, the decision to stop antiplatelets were individualized. A treatment effectiveness analysis was also performed, trichotomizing the patient group to AC and AP groups that adhered to the original treatment, and a third group of antithrombotic cross-overs from AC to AP. The main analysis of the study was performed in a treatment policy analysis basis.

### Clinical variables

The patient’s clinical presentation was dichotomized to ‘ischemic stroke and transient ischemic attack (TIA)’ or ‘headache and others.’ The presence of headache as an accompanying symptom was also analyzed (13). The arterial luminal morphology of the dissecting segment was described as pure steno-occlusive patterns or dilatation patterns (including stenosis and dilatation) (14). The dissection location was categorized as anterior or posterior circulation. New ischemic stroke or hemorrhagic stroke events that occurred within the 3-month period after patient’s primary presentation were identified through review of medical records. As recurrent ischemic stroke events are known to predominate in the early (1 to 7 days) phases (3), we also evaluated early neurological deterioration (END, defined as an increase in the National Institute of Health Stroke Scale score by 2 or more points within 7 days post-admission (15)) related to cerebral ischemia. The primary clinical endpoint was a composite of clinical outcomes (combination of ischemic END, recurrent ischemic or hemorrhagic stroke, or death which occurred within 3 months).

### Imaging variables and endovascular reconstructive/deconstructive therapy

In unruptured IAD patients with risk for rupture, endovascular reconstructive or deconstructive therapy (5) was performed by flow diversion via stent within stent technique (16) or embolization via endovascular coiling/stent-assisted coiling procedures. It was performed by attending physicians’ discretion with consensus especially on vertebrobasilar IAD with a diameter ratio between dissecting and normal segment of the artery of ≥1.5 or significant progression of dissection (17).

The arterial imaging and diagnosis of IAD was based on combined imaging findings of CT angiography, transfemoral cerebral angiography, or high resolution-magnetic resonance imaging. Serial angiographic images were analyzed to evaluate luminal arterial changes. Arterial changes were usually interpreted based on non-invasive angiographic images, more commonly CT based than MR based. We dichotomized arterial changes to early (during primary hospitalization) and late (after discharge) time frames. This dichotomization was used for three reasons. First, as rupture of dissecting aneurysms is known to predominate in the early phases of no more than 2 weeks (18), arterial changes may also dominate in this period. Second, while antithrombotics may be selected by decision to perform endovascular treatment (e.g., AP pretreatment for flow diversion via stents or stent-assisted coiling), early arterial changes may in turn call for endovascular treatment. Third, as this is a retrospective study, we presumed that concern about urgent arterial changes would have been resolved to some extent at the time of discharge.

In the early time frame, aneurysmal changes (new appearance of aneurysm or increase in aneurysm size), presence of recanalization of a previous occlusive segment, or overall combined arterial changes, were independently evaluated. In the late (after discharge) time frame, along with late aneurysmal changes, overall arterial healing, defined as normalization or improvement in the luminal diameter for stenotic or occlusive lesions and normalization or decrease in aneurysm size for dilatation patterns (14), were evaluated to represent overall directionality of the vascular healing process.

### Statistical analysis

First, in patients that presented with symptomatic unruptured IAD, patients that were managed with AC and AP therapy were compared for primary clinical and arterial endpoints based on treatment policy analysis. A 1:1 propensity score matching (PSM) was further performed to correct for baseline imbalances. Second, patients that were managed with AC and AP therapy were compared based on a treatment effectiveness analysis, also with PSM. A standardized difference of less than 0.2 was considered acceptable. Cross-overs from AC to AP were also analyzed.

Continuous variables were compared using Student’s t-test, or Kruskal Wallis test. Categorical variables were analyzed using the chi-square test or Fisher’s exact test. All statistical analyses were performed using IBM SPSS (version 25.0 for Windows, IBM Corp., Armonk, NY, USA). Statistical significance was set at *p* < 0.05.

## Results

### Treatment policy analysis

A detailed flowchart of the patients included in the current study is shown in Figure 1. A total of 311 patients were included in the analysis. Among 311 unruptured IAD patients, AC was used in 211 (67.8%) while AP was used in 100 (32.2%). In the AP group, dual antiplatelets were used in 45 (45.0%). While single antiplatelet was used in 55 (55.0%). When the two groups were compared (Table 1), the AC group more frequently presented with ischemic stroke and transient ischemic attacks (74.4% vs. 51.0%, *p* < 0.001), steno-occlusive morphology (63.0% vs. 39.0%, *p* < 0.001), male sex (71.6% vs. 60.0%, *p* = 0.041), and higher rates of comorbid hypertension (40.8% vs. 26.0%, *p* = 0.011) compared to AP group. For the primary endpoint at 3 months, there were no differences between the two groups (11.8% vs. 7.0%, *p* = 0.193). In detail, there were no differences for ischemic END, new ischemic stroke, new SAH or ICH, or mortality at 3 months. A detailed analysis of the chief complaints, involved arteries, and dissecting luminal morphology is presented in Supplementary Table 1. Detailed analysis of the diagnostic imaging modalities utilized is presented in Supplementary Table 2.

**Table 1:** Comparison of baseline characteristics and outcomes of antithrombotic therapy in intracranial arterial dissections (treatment policy analysis).

	Original			PSM		
	AC (*N* = 211)	AP (*N* = 100)	*p*	AC (*N* = 99)	AP (*N* = 99)	*p*
Baseline characteristics						
Age	47 [41–53]	49.5 [43–55]	0.093	51 [54–55]	50 [43–55]	0.715
Onset-to-presentation, d	2 [0–6]	3 [1–7]	0.167	3 [1–8]	3 [1–7]	0.632
Ischemic stroke & TIA	157 (74.4%)	51 (51.0%)	<0.001	55 (55.6%)	50 (50.5%)	0.476
NIHSS	2.0 [0.0–4.0]	1.0 [0.0–4.25]	0.193	1.0 [0.0–4.0]	1.0 [0.0–4.25]	0.384
Headache	150 (71.1%)	73 (73.0%)	0.727	74 (74.7%)	72 (72.7%)	0.747
Sex, male	151 (71.6%)	60 (60.0%)	0.041	63 (63.6%)	59 (59.6%)	0.559
Morphology			<0.001			0.884
Steno-occlusive	133 (63.0%)	39 (39.0%)		37 (37.4%)	38 (38.4%)	
Dilatation	78 (37.0%)	61 (61.0%)		62 (62.6%)	61 (61.6%)	
Posterior circulation	185 (87.7%)	84 (84.0%)	0.375	83 (83.8%)	83 (83.8%)	0.99
HTN	86 (40.8%)	26 (26.0%)	0.011	33 (33.3%)	26 (26.3%)	0.277
DM	15 (7.1%)	10 (10.0%)	0.381	3 (3.0%)	10 (10.1%)	0.045
Smoking	74 (35.1%)	35 (35.0%)	0.990	32 (32.3%)	34 (34.3%)	0.763
Dyslipidemia	45 (21.3%)	16 (16.0%)	0.269	16 (16.2%)	16 (16.2%)	0.99
Early vascular outcomes						
Early endovascular reconstructive/deconstructive treatment	5 (2.4%)	18 (18.0%)	<0.001	4 (4.0%)	18 (18.2%)	0.002
Recanalization	18/209 (8.6%)	7/94 (7.4%)	0.733	7/98 (7.1%)	7/93 (7.5%)	0.919
Early aneurysmal change	17/209 (8.1%)	8/94 (8.5%)	0.912	9/98 (9.2%)	8/93 (8.6%)	0.888
Early arterial changes	35/209 (16.7%)	17/94 (18.1%)	0.775	17/98 (17.3%)	17/93 (18.3%)	0.866
Clinical outcomes						
Primary endpoint at 3 months	25/212 (11.8%)	7/100 (7.0%)	0.193	9/96 (9.4%)	6/92 (6.5%)	0.470
Ischemic END	14 (6.6%)	4/100 (4.0%)	0.353	5/99 (5.1%)	4/99 (4.0%)	0.733
New ischemic stroke	10/207 (4.8%)	1/94 (1.1%)	0.106	3/96 (3.1%)	1/93 (1.1%)	0.328
New SAH or ICH	2/207 (1.0%)	1/93 (1.1%)	0.930	1/96 (1.0%)	1/92 (1.1%)	0.976
Mortality at 3 months	1/205 (0.5%)	2/92 (2.2%)	0.179	1/94 (1.1%)	2/91 (2.2%)	0.542
Late vascular outcomes						
Late endovascular reconstructive/deconstructive treatment	2/206 (1.0%)	4/82 (4.9%)	0.036	2/95 (2.1%)	4/81 (4.9%)	0.302
Late aneurysmal change	10/179 (5.6%)	6/62 (9.7%)	0.265	5/83 (6.0%)	6/61 (9.8%)	0.395
Arterial healing	103/183 (57.9%)	35/62 (56.5%)	0.846	51/83 (61.4%)	35/61 (57.4%)	0.623

The data are presented as the median [quartiles], or number (%) as appropriate.

PSM, propensity score matching; AC, anticoagulation; AP, antiplatelets; TIA, transient ischemic attack; NIHSS, National Institute of Health Stroke Scale; HTN, hypertension; DM, diabetes mellitus; END, early neurological deterioration; SAH, subarachnoid hemorrhage; ICH, intracerebral hemorrhage.

In terms of early vascular outcomes, there was a higher number of early endovascular repair performed in the AP group (2.4% vs. 18.0%, *p* < 0.001). However, there were no differences in the rates of recanalization (8.6% vs. 7.4%, *p* = 0.775), early aneurysmal changes (8.1% vs. 8.5%, *p* = 0.912), or combined early arterial changes (16.7% vs. 18.1%, 0.775).

For late vascular outcomes, while there was a higher rate of late endovascular repair in the AP group (1.0% vs. 4.9%, *p* = 0.036), there were no differences in late aneurysmal changes (5.6% vs. 9.7%, *p* = 0.265) or overall arterial healing (57.9% vs. 56.5%, *p* = 0.846).

### Treatment policy analysis: propensity score matched

Due to differences in baseline characteristics, a 1:1 propensity score matching was performed to correct for age, ischemic presentation, morphology, and anterior vs. posterior circulation (Table 1). After matching, all differences in baseline characteristics between the groups have been corrected. For the primary endpoint at 3 months, there were no differences between the two groups (9.4% vs. 6.5%, *p* = 0.470). In detail, there were no differences for ischemic END, new ischemic stroke, new SAH or ICH, or mortality at 3 months.

In early vascular outcomes, there was a higher rate of early endovascular repair in the AP group (4.0% vs. 18.2%, *p* = 0.002). However, there were no differences in the rates of recanalization (7.1% vs. 7.5%, *p* = 0.919), early aneurysmal changes (9.2% vs. 8.6%, *p* = 0.888), or combined early arterial changes (17.3% vs. 18.3%, 0.866).

For late vascular outcomes, there were no differences in rate of late endovascular reconstructive or deconstructive treatment (2.1% vs. 4.9%, *p* = 0.302), late aneurysmal changes (6.0% vs. 9.8%, *p* = 0.395), or overall arterial healing (61.4% vs. 57.4%, *p* = 0.623).

### Treatment effectiveness analysis and cross-over group

Among 211 patients with AC, there were 57 crossovers to AP (57/211, 27.0%). The results of the treatment effectiveness analysis that compares AC and AP groups resemble that of treatment policy analysis before and after propensity score matching (Supplementary Table 3).

As the cross-over group is a special population worth attention, the differences between the AC group and the cross-over group were compared (Table 2). There were no differences in baseline characteristics between the two groups. A higher rate of early endovascular reconstructive or deconstructive treatment was seen in the cross-over group (0.6% vs. 7.0%, *p* = 0.007), this time supported by a higher rate of early arterial changes (13.1% vs. 26.8%, *p* = 0.019) including early aneurysmal changes (5.2% vs. 16.1%, *p* = 0.011) and early recanalization (5.2% vs. 17.9%, *p* = 0.004) in this specific group. The difference for early arterial changes (odds ratio: 2.42, 95% confidence interval [1.11–5.25], *p* = 0.025), early aneurysmal changes (OR: 3.76, 95% CI [1.26–11.16], *p* = 0.017) and early recanalization (OR: 4.08 [1.47–11.29], *p* = 0.007) was significant after correcting for age, sex, and arterial morphology. Afterwards, there were no differences in primary endpoint at 3 months, clinical outcomes, or late vascular outcomes.

**Table 2:** Comparison of baseline characteristics and outcomes of Anticoagulation group and cross-over to antiplatelet group in intracranial arterial dissections (treatment effectiveness analysis).

	AC (*N* = 154)	Cross-over to AP (*N* = 57)	*p*
Baseline characteristics			
Age	47 [40–53]	48 [41.5–53.5]	0.179
Onset-to-presentation, d	2 [0–6.25]	1 [0–6]	0.246
Ischemic stroke & TIA	110 (71.4%)	47 (82.5%)	0.103
NIHSS	2.0 [0.0–4.0]	1.0 [0.0–3.0]	0.033
Headache	110 (71.4%)	40 (70.2%)	0.858
Sex, male	110 (71.4%)	41 (71.9%)	0.943
Morphology			0.765
Steno-occlusive	99 (63.6%)	35 (61.4%)	
Dilatation	56 (36.4%)	22 (38.6%)	
Posterior circulation	134 (87.0%)	51 (89.5%)	0.629
HTN	61 (39.6%)	25 (43.9%)	0.577
DM	9 (5.8%)	6 (10.5%)	0.240
Smoking	58 (37.7%)	16 (28.1%)	0.195
Dyslipidemia	32 (20.8%)	13 (22.8%)	0.749
Early vascular outcomes			
Early endovascular reconstructive/deconstructive treatment	1 (0.6%)	4 (7.0%)	0.007
Early recanalization	8/153 (5.2%)	10/56 (17.9%)	0.004
Early aneurysmal change	8/153 (5.2%)	9/56 (16.1%)	0.011
Early arterial changes	20/153 (13.1%)	15/56 (26.8%)	0.019
Clinical outcomes			
Primary endpoint at 3 months	17/150 (11.3%)	8/57 (14.0%)	0.594
Ischemic END	10 (6.3%)	4 (7.0%)	0.892
New ischemic stroke	6/151 (4.0%)	4/56 (7.1%)	0.345
New SAH or ICH	2/151 (1.3%)	0/56 (0.0%)	0.387
Mortality at 3 months	1/148 (0.7%)	0/57 (0.0%)	0.534
Late vascular outcomes			
Late endovascular reconstructive/deconstructive treatment	1/153 (0.7%)	1/53 (1.9%)	0.430
Late aneurysmal change	8/133 (6.0%)	2/46 (4.3%)	0.671
Arterial healing	78/133 (58.6%)	25/45 (55.6%)	0.717

The data are presented as the median [quartiles], or number (%) as appropriate.

AC, anticoagulation; AP, antiplatelets; TIA, transient ischemic attack; NIHSS, National Institute of Health Stroke Scale; HTN, hypertension; DM, diabetes mellitus; END, early neurological deterioration; SAH, subarachnoid hemorrhage; ICH, intracerebral hemorrhage.

### Endovascular reconstructive/deconstructive treatment for unruptured IAD

In the AP group, early endovascular reconstructive/deconstructive treatment was performed in 18 patients. Among them, flow diversion via stents were performed in 14, coil embolization was performed in 3, and stent-assisted coiling was performed in one. Late endovascular treatment was performed in 4 patients. In 3 patients, flow diversion via stents was performed, and coil embolization was performed in 1 patient.

In the AC group, early endovascular reconstructive/deconstructive treatment was performed in 5 patients. Coil embolization was performed in 3, and flow diversion via stents was performed in 2. In four of five patients, antithrombotics were changed to AP, then endovascular treatment was performed, or AP after procedure. Two patients underwent late endovascular treatment. In one patient under oral AC, occlusive vertebrobasilar dissecting aneurysm underwent large aneurysmal change with recanalization at the third month and coil embolization was performed. In one patient, AC was shifted to AP before discharge. Flow diversion via stents was performed afterwards.

### Detailed review of subsequent hemorrhages

One case of SAH and one cerebellar hemorrhage (possibly anticoagulation related) occurred in the AC group, while one case of SAH occurred in the AP group. Both SAH cases occurred within 5 days of symptom onset. The case in the AC group presented with headaches and showed a purely dilatation morphology dissection at the V4 portion with a diameter ratio between dissecting and normal segment less than 1.5 (Supplementary Figure 1A). While IV heparin was used to prevent thrombotic complications, rupture occurred on the next hospital day. The case in the AP group showed an atypical course in a patient with liver cirrhosis (Supplementary Figure 1B). The patient presented with oculomotor nerve palsy due to distal ICA dissection. Bilateral blindness due to optic ischemia occurred the next day, followed by SAH. SAH was unexpected in this patient because of the lack of arterial dilatation and early rupture. Atypical disease course led us to suspect vasculitis, but autopsy was not performed.

## Conclusion

The current study results showed no differences in clinical outcomes evaluated as a 3-month composite primary endpoint between AC and AP for symptomatic unruptured IAD patients. A higher rate of early and late endovascular reconstructive/deconstructive treatment in the AP group was seen, but it was not supported by differences in early and late vascular outcomes, most likely representing predilection to use AP before endovascular repair. However, crossover from AC to AP was not infrequent, and early recanalization and aneurysmal changes were more common in the crossover group, likely resulting in the crossover of antithrombotic regimen. While the best antithrombotic regimen is still unclear and needs future studies, tailored antithrombotic regimens may be used for IAD with serial angiographic imaging to achieve optimal results.

In detailed analysis of primary endpoints, there were no differences in ischemic stroke related outcomes according to antithrombotic choice. Both the overall rate of ischemic END (5.8%) and ischemic stroke (3.5%) were low in the current study, and comparable to randomized controlled trials of CAD, which failed to show differences in outcomes between AC and AP. The CADISS trial recruited patients in whom ischemic stroke was present in 77.6%, and reported a 5.2% rate of any stroke or TIA at 1 year, and 2.4% rate of ipsilateral stroke at 1 year (2). The TREAT-CAD trial recruited patients in whom ischemic stroke was present in 52.1%, and reported a 3.6% rate of ischemic stroke at 3 months (3). To our knowledge, the current study used the largest dataset to compare the results of AC and AP therapy for IAD. While the current registry is retrospective in design and underpowered due to low ischemic events to draw conclusions regarding antithrombotics and ischemic outcomes, based on the current results, it is likely that the absolute differential effect of AC and AP on ischemic outcome would be very low.

In unruptured IAD, there is risk for subsequent rupture and SAH unlike CAD, which can be devastating. In this study, apart from a higher rate of early and late endovascular reconstructive/deconstructive treatment performed in the AP group, there were no differences in arterial changes, or new SAH or ICH according to antithrombotics. The higher rate of endovascular reconstructive/deconstructive treatment in the AP group is reasonable, as antiplatelets may have been used prior to flow diverter stents or stent-assisted coiling. Two cases of SAH occurred during admission, showing that rupture of dissecting aneurysms predominate in the early phase of dissections (18). Furthermore, their location of dissection was V4 segment of the vertebral artery and the intracranial ICA, possibly showing predilection for rupture to occur in areas of transition from cervical to intracranial arteries occur (7). Apart from this, subsequent hemorrhage was rare. It should be noted that while early aneurysmal changes were overall numerically more frequent, late aneurysmal changes were overall not uncommon, demonstrating the importance of repeat imaging.

Considering the devastating nature of aneurysmal rupture, for unruptured IADs that do not present with ischemia, thrombotic risk should be stratified, and AC, or possibly antithrombotics, may be avoided in populations with low thrombotic risk. One example could be pure dilatation morphologies of dissections. It is generally accepted that steno-occlusive luminal morphology (8) is associated with ischemic risk in IAD. However, as a recent study claimed that stenotic segments more frequently occur in ruptured IVAD (19), luminal morphology-based decisions may not be sufficient. We have recently shown that higher arterial pulse wave velocity is associated with ischemia (6). Another study showed that a proximal dominant intramural hematoma in comparison to a distal one is associated with ischemic stroke (20). Individualized antithrombotics selection may be tailored based on such knowledge. Antithrombotics may be also selected according to whether the intimal flap ruptures and whether flow patency is maintained. Dissections with intact intima with or without limited flow patency were reported to more frequently present with ischemic symptoms, while healing was more common (21). Such findings may guide antithrombotic therapy, while these findings have to be further confirmed in IAD.

While current guidelines advocate the use of AP over AC in IAD due to better risk/benefit ratio (4), the current results show that AC may be safely used according to the clinician’s discretion and considering the potential advantages of each modality. While the treatment policy analysis shows no difference between AC and AP in rates of early and late arterial changes other than endovascular reconstructive/deconstructive treatment, crossovers from AC to AP were not uncommon. There was a higher rate of early recanalization and aneurysmal changes in the crossover group, which was likely the cause of crossover. There may be situations in which a specific regimen is preferred in a case-by-case basis. First, AC may be more preferable in patients long dissecting segments or dissecting occlusions, possibly able to cause worse outcomes due to perfusion failure (22). Endovascular treatment may also be limited by risk of intraprocedural arterial rupture in such patients (23). Recanalization through AC may be helpful for these patients because there still may be minimal arterial flow, sparing the critical penumbra slower than embolic occlusions. Second, AP may be preferable to AC if there is risk for aneurysmal enlargement. We have experienced patients that underwent early aneurysmal enlargement while under AC, and switching to AP resulted in its regression. Supplementary Figure 2A shows an example of successful regression of the aneurysmal segment after switching to antiplatelet agents. If anticoagulation is used, high-resolution arterial imaging may aid arterial follow-up because it can visualize potential arterial positive remodeling and aneurysmal changes that may not be visualized due to thrombosed pseudolumen (24). In some cases, oral AC may aid late arterial healing of stenosis of arterial dissecting segments (Supplementary Figure 2B).

The current study is inherently limited by the retrospective study design. First, baseline differences were observed between those with AC and AP, with higher number of ischemic stroke and TIA presentations, steno-occlusive arterial morphology, and vascular risk factor in the AC group. In some patient that presented with unruptured VAD, antiplatelets may have seen used as a premedication prior to flow diverter stenting or stent-assisted coiling (25, 26). Given this, the subsequent risk of ischemia may be biased to be higher in the AC group. Propensity score matching was performed in this regard. Patients that underwent mechanical thrombectomy or those that did not use antithrombotics were also excluded from the current study, as these patients would usually present with large infarcts and clinicians tend to avoid using AC in this population. After excluding such patients and propensity score matching, there were no differences in composite clinical outcomes between AC and AP groups. Second, the primary composite clinical outcome is prone to bias, as patients were treated to reduce hemorrhagic complications or ischemic complications in an individual basis. Thus, the differences in treatment outcomes according to thrombotic regimens are expected to decay. The high rate of transition in medical therapies is an example. Meanwhile, we believe that the current study results can guide clinicians through pros and cons of individualized antithrombotic therapy for IAD. Third, the description of arterial outcomes may be rather subjective, as various angiographic modalities were used. The outcome arterial healing in late vascular outcomes may be subjective, as it encompasses a wide range of arterial improvements such as normalization to mild improvements in luminal diameter. Small changes in appearance may be affected by different gantry angles and slice thicknesses. In the current study, as aneurysmal enlargement or recanalization were separately evaluated, arterial healing was used as a more inclusive terminology to represent the overall direction of vascular repair processes.

In conclusion, in an observational study of symptomatic IAD patients, there were no differences in composite clinical outcomes between AC and AP. Crossovers from AC to AP was common and associated with early vascular changes. Both AC and AP may be tailored based on clinician’s decision and repeat arterial imaging.

## Data Availability

Data supporting the findings of this study are available from the corresponding author upon reasonable request.
