# Supplementary material for: Comparison of outcomes between anticoagulation and antiplatelet therapies for intracranial arterial dissections
Source: Front Neurol. 2024 Dec 4;15:1469697. doi: 10.3389/fneur.2024.1469697 (PMC11652506; doi:10.3389/fneur.2024.1469697)
Supplement: Supplementary file 1 [file Data_Sheet_1.pdf]

Comparison of clinical and imaging outcomes between anticoagulation and antiplatelet therapies for intracranial arterial dissections

Seong-Joon Lee[1], Jin Soo Lee[1], Min Kim[1], So Young Park[1], Ji Hyun Park[2], BumHee Park[23], Woo Sang Jung[4], Jin Wook Choi[4], Yong Cheol Lim[5], Ji Man Hong[1]

[1]Department of Neurology, Ajou University School of Medicine, Suwon, Republic of Korea

[2]Office of Biostatistics, Medical Research Collaborating Center, Ajou Research Institute for Innovative Medicine, Ajou University Medical Center, Suwon, Republic of Korea

[3]Department of Biomedical Informatics, Ajou University School of Medicine, Suwon, Republic of Korea

[4]Department of Radiology, Ajou University School of Medicine, Suwon, Republic of Korea.

[5]Department of Neurosurgery, Ajou University School of Medicine, Suwon, Republic of Korea.

Correspondence to:

Jin Soo Lee

Supplementary Table 1. Detailed analyses of chief complaints, involved arteries, and dissecting segment luminal morphology.

	Original			PSM		
	AC (N=154)	AP (N=99)	P	AC (N=97)	AP (N=97)	P
**Chief complaint**						
Mental change	5 (2.4%)	3 (3.0%)	0.015	3 (3.0%)	3 (3.0%)	0.335
weakness	45 (21.3%)	15 (15.0%)		16 (16.2%)	15 (15.2%)	
Ataxia/cerebellar	34 (16.1%)	7 (7.0%)		15 (15.2%)	6 (6.1%)	
Other neurological deficits	8 (3.8%)	5 (5.0%)		2 (2.0%)	5 (5.1%)	
Headache	63 (29.9%)	50 (50.0%)		47 (47.5%)	50 (50.5%)	
Dizziness	53 (25.1%)	20 (20.0%)		16 (16.2%)	20 (20.2%)	
Others	3 (1.4%)	0 (0.0%)		0 (0.0%)	0 (0.0%)	
**Involved arteries**						
Anterior circulation			0.078			0.497
ICA	4 (1.9%)	4 (4.0%)		2 (2.0%)	4 (4.0%)	
MCA	15 (7.1%)	9 (9.0%)		8 (8.1%)	9 (9.1%)	
ACA	5 (2.4%)	3 (3.0%)		5 (5.1%)	3 (3.0%)	
Combined extra and intracranial	2 (0.9%)	0 (0.0%)		1 (1.0%)	0 (0.0%)	
Posterior circulation						
VA	93 (44.1%)	57 (57.0%)		58 (58.6%)	57 (57.6%)	
BA	7 (3.3%)	4 (4.0%)		2 (2.0%)	4 (4.0%)	

VBA	18 (8.5%)	2 (2.0%)		6 (6.1%)	2 (2.0%)	
PCA	2 (0.9%)	3 (3.0%)		0 (0.0%)	3 (3.0%)	
PICA	26 (12.3%)	8 (8.0%)		9 (9.1%)	7 (7.1%)	
Combined extra and intracranial	39 (18.5%)	10 (10.0%)		8 (8.1%)	10 (10.1%)	
**Luminal morphology**						
**Steno-occlusive**			<0.001			0.135
Stenosis	59 (28.0%)	18 (18.0%)		17 (17.2%)	17 (17.2%)	
Multifocal stenosis	17 (8.1%)	6 (6.0%)		3 (3.0%)	6 (6.1%)	
Occlusion	43 (20.4%)	12 (12.0%)		13 (13.1%)	12 (12.1%)	
Combined occlusion and stenosis&dilatation	8 (3.8%)	3 (3.0%)		2 (2.0%)	3 (3.0%)	
Multifocal steno-occlusion	6 (3.8%)	0 (0.0%)		2 (2.0%)	0 (0.0%)	
**Dilatations**						
Dilatation	16 (7.6%)	27 (27.0%)		14 (14.1%)	27 (27.3%)	
Stenosis&dilatation	62 (29.4%)	34 (34.0%)		48 (48.5%)	34 (34.3%)	
**Late vascular outcomes**						
Late endovascular reconstructive/deconstructive treatment	1/153 (0.7%)	4/82 (4.9%)	0.032	1/96 (1.0%)	4/80 (5.0%)	0.116
Late aneurysmal change	8/133 (6.0%)	6/62 (9.7%)	0.356	6/86 (7.0%)	6/61 (9.8%)	0.533
Arterial healing	78/133 (58.6%)	35/62 (56.5%)	0.772	53/85 (62.4%)	35/61 (57.4%)	0.545

PSM, propensity score matching; AC, anticoagulation; AP, antiplatelets; ICA, internal carotid artery; MCA, middle cerebral artery; ACA,

anterior cerebral artery; VA, vertebral artery; BA, basilar artery; VBA, vertebra-basilar artery; PCA, posterior cerebral artery; PICA, posterior inferior cerebellar artery.

Supplementary Table 2. Diagnostic imaging modalities utilized.

	Original			PSM		
	**AC (N=211)**	**AP (N=100)**	**P**	**AC (N=99)**	**AP (N=99)**	**P**
CTA	206 (97.6%)	98 (98.0%)	0.837	96 (97.0%)	97 (98.0%)	0.651
TFCA	150 (71.1%)	66 (66.0%)	0.363	75 (75.8%)	66 (66.7%)	0.158
HR-MRI	194 (91.9%)	79 (79.0%)	0.001	91 (91.9%)	78 (78.8%)	0.009

PSM, propensity score matching; AC, anticoagulation; AP, antiplatelets; CTA, computed tomography angiography; TFCA, transfemoral cerebral angiography; HR-MRI, high resolution magnetic resonance imaging.

Supplementary Table 3. Comparison of baseline characteristics and outcomes of antithrombotic therapy in intracranial arterial dissections (treatment effectiveness analysis).

	Original			PSM		
	AC (N=154)	AP (N=99)	P	AC (N=97)	AP (N=97)	P
**Baseline characteristics**						
Age	47 [40 – 53]	49.5 [43 – 55]	0.052	49 [42 – 54]	49 [43 – 55]	0.742
Onset-to-presentation, d	2.0 [0.0 – 6.25]	3.0 [1.0 – 7.0]	0.370	3.0 [0.0 – 7.5]	3.0 [1.0 – 7.0]	0.818
Ischemic stroke & TIA	110 (71.4%)	50 (50.5%)	0.001	59 (60.8%)	50 (51.5%)	0.193
NIHSS	2.0 [0.0 – 4.0]	1.0 [0.0 – 4.0]	0.469	2.0 [0.0 – 4.0]	1.0 [0.0 – 4.25]	0.727
Headache	110 (71.4%)	73 (73.0%)	0.785	72 (74.2%)	70 (72.2%)	0.746
Sex, male	110 (71.4%)	60 (60.0%)	0.059	63 (64.9%)	59 (60.8%)	0.552
Morphology			<0.001			0.383
Steno-occlusive	98 (63.6%)	39 (39.0%)		44 (45.4%)	38 (39.2%)	
Dilatation	56 (36.4%)	61 (61.0%)		53 (54.6%)	59 (60.8%)	
Posterior circulation	134 (87.0%)	84 (84.0%)	0.501	79 (81.4%)	81 (83.5%)	0.705
HTN	61 (39.6%)	26 (26.0%)	0.026	32 (32.0%)	26 (26.8%)	0.347
DM	9 (5.8%)	10 (10.0%)	0.219	5 (5.2%)	10 (10.3%)	0.179
Smoking	58 (37.7%)	35 (35.0%)	0.667	37 (38.1%)	34 (35.1%)	0.655
Dyslipidemia	32 (20.8%)	16 (16.0%)	0.342	15 (15.5%)	16 (16.5%)	0.845

**Early vascular outcomes**						
Early endovascular reconstructive/deconstructive treatment	1 (0.6%)	18 (18.0%)	<0.001	1 (1.0%)	17 (17.5%)	<0.001
Early recanalization	8/153 (5.2%)	7/94 (7.4%)	0.479	5/96 (5.2%)	7/91 (7.7%)	0.488
Early aneurysmal change	8/153 (5.2%)	8/94 (8.5%)	0.309	6/96 (6.3%)	8/91 (8.8%)	0.509
Early arterial changes	20/153 (13.1%)	17/94 (18.7%)	0.284	13/96 (13.5%)	17/91 (18.7%)	0.338
**Clinical outcomes**						
**Primary endpoint at 3 months**	17/150 (11.3%)	6/93 (6.5%)	0.206	9/94 (9.6%)	6/90 (6.7%)	0.471
Ischemic END	10 (6.5%)	4 (4.0%)	0.395	5 (5.2%)	4 (4.1%)	0.733
New ischemic stroke	6/151 (4.0%)	1 (1.1%)	0.184	4/94 (4.3%)	1/91 (1.1%)	0.186
New SAH or ICH	2/151 (1.3%)	1/93 (1.1%)	0.864	1/94 (1.1%)	1/90 (1.1%)	0.975
Mortality at 3 months	1/148 (0.7%)	2/92 (2.2%)	0.310	1/92 (1.1%)	2/89 (2.2%)	0.541
**Late vascular outcomes**						
Late endovascular reconstructive/deconstructive treatment	1/153 (0.7%)	4/82 (4.9%)	0.032	1/96 (1.0%)	4/80 (5.0%)	0.116
Late aneurysmal change	8/133 (6.0%)	6/62 (9.7%)	0.356	6/86 (7.0%)	6/61 (9.8%)	0.533
Arterial healing	78/133 (58.6%)	35/62 (56.5%)	0.772	53/85 (62.4%)	35/61 (57.4%)	0.545

The data are presented as the median [quartile], or number (%) as appropriate.

PSM, propensity score matching; AC, anticoagulation; AP, antiplatelets; TIA, transient ischemic attack; NIHSS, National Institute of Health Stroke Scale; HTN, hypertension; DM, diabetes mellitus; END, early neurological deterioration; SAH, subarachnoid hemorrhage; ICH,

intracerebral hemorrhage.

Supplementary Figure 1. Two subarachnoid hemorrhage events occurred after hospitalization. A. A female patient in her fifties without any underlying disease presented with headache. CT angiography showed IAD of the intracranial VA with pure dilatation luminal morphology. Positive remodeling of the outer arterial wall, dissection flap, and perivascular enhancement was seen in HR-MRI. IV heparin was administered. However, the next day, the patient became comatose after a sudden onset headache, and subarachnoid hemorrhage occurred, with slight enlargement of the dissecting aneurysm. **B.** A male in his sixties with underlying liver cirrhosis presented with diplopia, localizing as left oculomotor nerve paly. Brain MRI, and CT angiography findings were unrevealing, and the patient was hospitalized. However, the patient experienced sudden visual loss, and repeat brain MRI/A revealed multifocal irregular stenosis of the ICA petrous segment, suspicious dilatation of the intracranial ICA, and diffusion restriction of the left optic nerve. Subsequently, the patient suddenly became comatose with subarachnoid hemorrhage due to rupture of the dilated intracranial ICA segment.

CT, computed tomography; IAD, intracranial arterial dissections; HR-MRI, high resolution magnetic resonance imaging; IV, intravenous; MRI, magnetic resonance imaging; ICA, internal carotid artery.

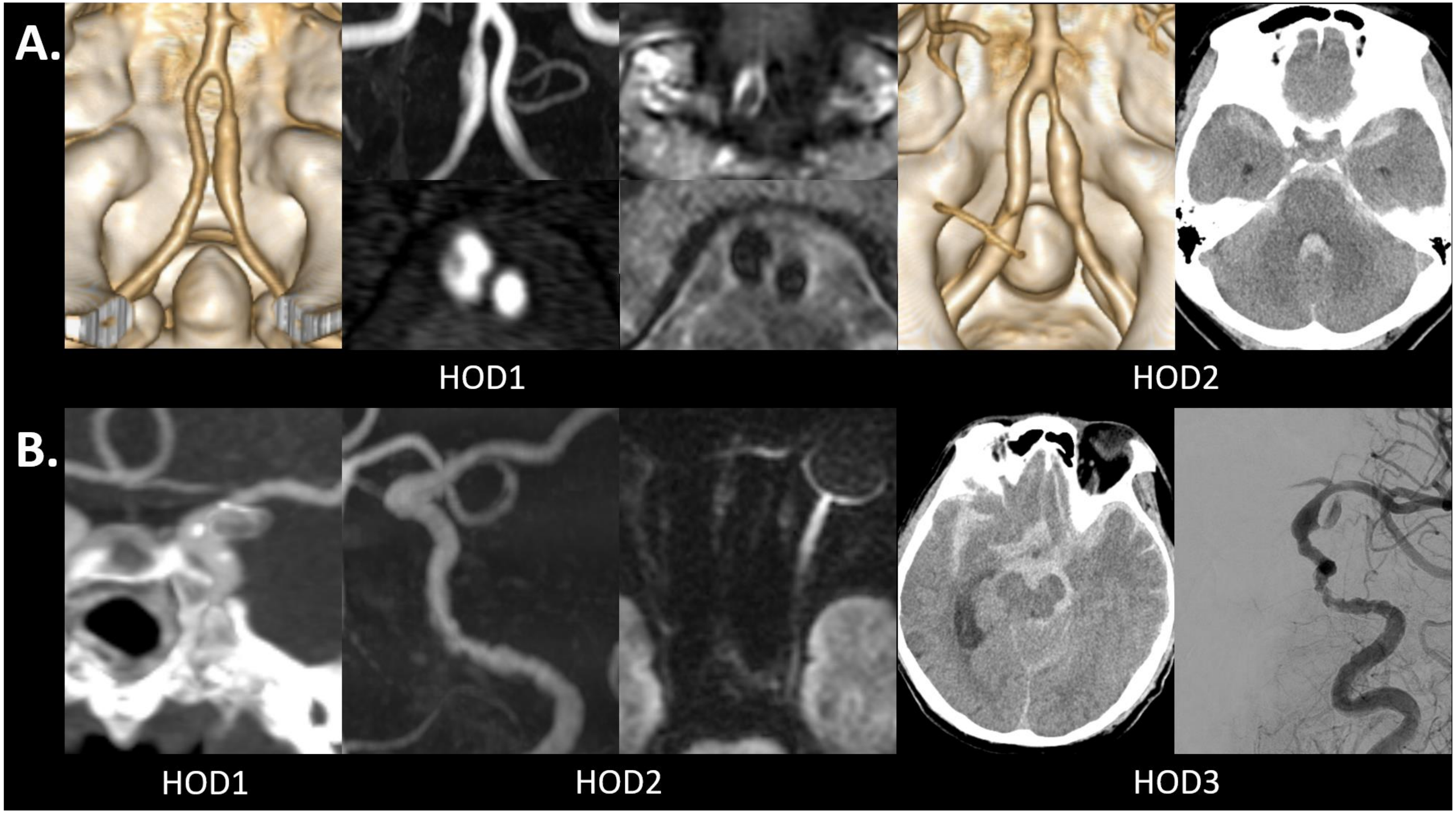
A.
HOD1
HOD2
B.
HOD1
HOD2
HOD3

Supplementary Figure 2. Example cases of dynamic vascular changes associated with anticoagulation. A. A male in his thirties presented with dizziness and vertigo. CT angiography showed bilateral dissecting occlusion of the vertebral arteries, and IV heparin was started. HR MRI performed the next day showed recanalization of the right vertebral artery, adjacent intramural hematoma, and positive remodeling of the outer arterial wall. On the 4th hospital day, the arterial recanalization was obvious, but aneurysmal transformation occurred, which enlarged up to the eleventh hospital day. After discontinuing anticoagulation, the dissecting aneurysm normalized. **B.** A male in his thirties experienced thunderclap headache. Brain MRI showed severe focal stenosis of distal ICA and its bifurcation. He was transferred to our hospital, and CT angiography showed improvement of the ICA bifurcation stenosis, with residual mild stenosis of the distal ICA, and enhancement of arterial wall on HR MRI. While no flap was visualized, arterial dissection could be diagnosed based on the clinical presentation and rapid changes in arterial morphology. Antiplatelet therapy was used. However, upon follow-up imaging, the arterial stenosis was aggravated. After transitioning to oral anticoagulation for 3 months, there was significant improvement of the arterial stenosis.

CT, computed tomography; IV, intravenous; MRI, magnetic resonance imaging; ICA, internal carotid artery; HR-MRI, high resolution magnetic resonance imaging.

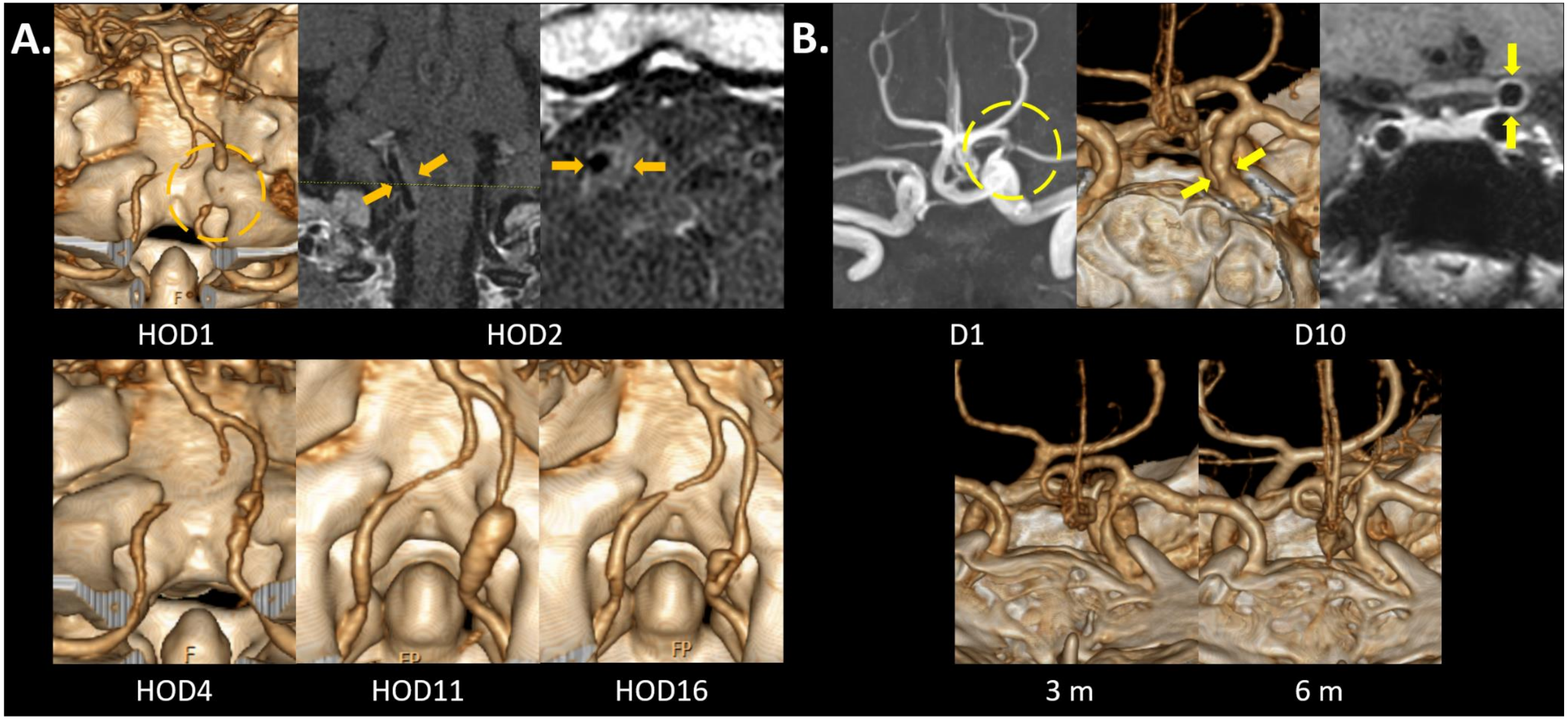
A.
HOD1
HOD2
HOD4
HOD11
HOD16
B.
D1
D10
3 m
6 m
